# Supplementary material for: Trajectory of immune evasion and cancer progression in hepatocellular carcinoma
Source: Nat Commun. 2022 Mar 17;13:1441. doi: 10.1038/s41467-022-29122-w (PMC8931110; doi:10.1038/s41467-022-29122-w)
Supplement: Supplementary file 1 — Supplementary Information [file 41467_2022_29122_MOESM1_ESM.pdf]

Supplementary Information

Trajectory of immune evasion and cancer progression in hepatocellular carcinoma

Phuong H.D. Nguyen,[1#] Martin Wasser,[1,2#] Chong Teik Tan,[3] Chun Jye Lim,[1] Hannah L.H. Lai,[4] Justine Jia Wen Seow,[4] Ramanuj DasGupta,[4] Cheryl Z.J. Phua,[4] Siming Ma,[4] Jicheng Yang,[2] Sheena D/O Suthen,[1] Wai Leong Tam,[4,5,6,7] Tony K.H. Lim,[2,8] Joe Yeong,[2,8,9] Wei Qiang Leow,[2,8] Yin Huei Pang,[10] Gwyneth Soon,[10] Tracy Jiezhen Loh,[8] Wei Keat Wan,[8] Chung Yip Chan,[2,11] Peng Chung Cheow,[2,11] Han Chong Toh,[2,12] Alfred Kow,[13] Yock Young Dan,[14] Juinn Huar Kam,[2,11] Shridhar Iyer,[13] Krishnakumar Madhavan,[13] Alexander Chung,[2,11] Glenn K. Bonney,[13] Brian K.P. Goh,[2,11] Naiyang Fu,[2] Victor C. Yu,[3] Weiwei Zhai,[4,15,16] Salvatore Albani,[1,2*] Pierce K.H. Chow,[11,17*] Valerie Chew.[1,2*]

[1]Translational Immunology Institute (TII), SingHealth-DukeNUS Academic Medical Centre, Singapore 169856
[2]Duke-Nus Medical School, Singapore 169857
[3]Department of Pharmacy, National University of Singapore, Singapore 117559
[4]Genome Institute of Singapore (GIS), Agency for Science, Technology and Research (A*STAR), Singapore 138672
[5]School of Biological Sciences, Nanyang Technological University, Singapore 637551
[6]Cancer Science Institute of Singapore, National University of Singapore, Singapore 117599
[7]Department of Biochemistry, Yong Loo Lin School of Medicine, National University of Singapore, Singapore 117596
[8]Department of Anatomical Pathology, Singapore General Hospital, Singapore 169856
[9]Institute of Molecular and Cell Biology (IMCB), Agency for Science, Technology and Research (A*STAR), Singapore 138673
[10]Department of Pathology, National University Hospital, Singapore 119074
[11]Department of Hepatopancreatobiliary and Transplant Surgery, Division of Surgery and Surgical Oncology, Singapore General Hospital and National Cancer Centre Singapore, Singapore 169608.
[12]Division of Medical Oncology, National Cancer Centre Singapore, Singapore 169610
[13]Division of Hepatobiliary & Pancreatic Surgery, Department of Surgery, University Surgical Cluster, National University Health System, Singapore 119074
[14]Department of Medicine. Yong Loo Lin School of Medicine. National University of Singapore, Singapore 117597
[15]Key Laboratory of Zoological Systematics and Evolution, Institute of Zoology, Chinese Academy of Sciences, Beijing, China 100107
[16]Center for Excellence in Animal Evolution and Genetics, Chinese Academy of Sciences, Kunming, Yunan, China 650223
[17]Academic Clinical Programme for Surgery, SingHealth Duke-NUS Academic Medical Centre (AMC), Singapore 169857
[#] Equally Contributing Authors

*Corresponding authors: Professor Salvatore Albani, Translational Immunology Institute (TII), SingHealth-DukeNUS Academic Medical Centre, Singapore 169856; salvo@duke-nus.edu.sg; Professor Pierce K H Chow, Department of Hepatopancreatobiliary and Transplant Surgery, Division of Surgery and Surgical Oncology, Singapore General Hospital and National Cancer Centre Singapore, Singapore 169608; pierce.chow@duke-nus.edu.sg and Dr Valerie Chew, Translational Immunology Institute (TII), SingHealth-DukeNUS Academic Medical Centre, Singapore 169856; valerie.chew@duke-nus.edu.sg

Table of Contents

This file includes Supplementary Figures 1-8 and Supplementary Tables 1-8.

Supplementary Figures……………………………………...3
Supplementary Tables.....................................…………14

Supplementary Figures:

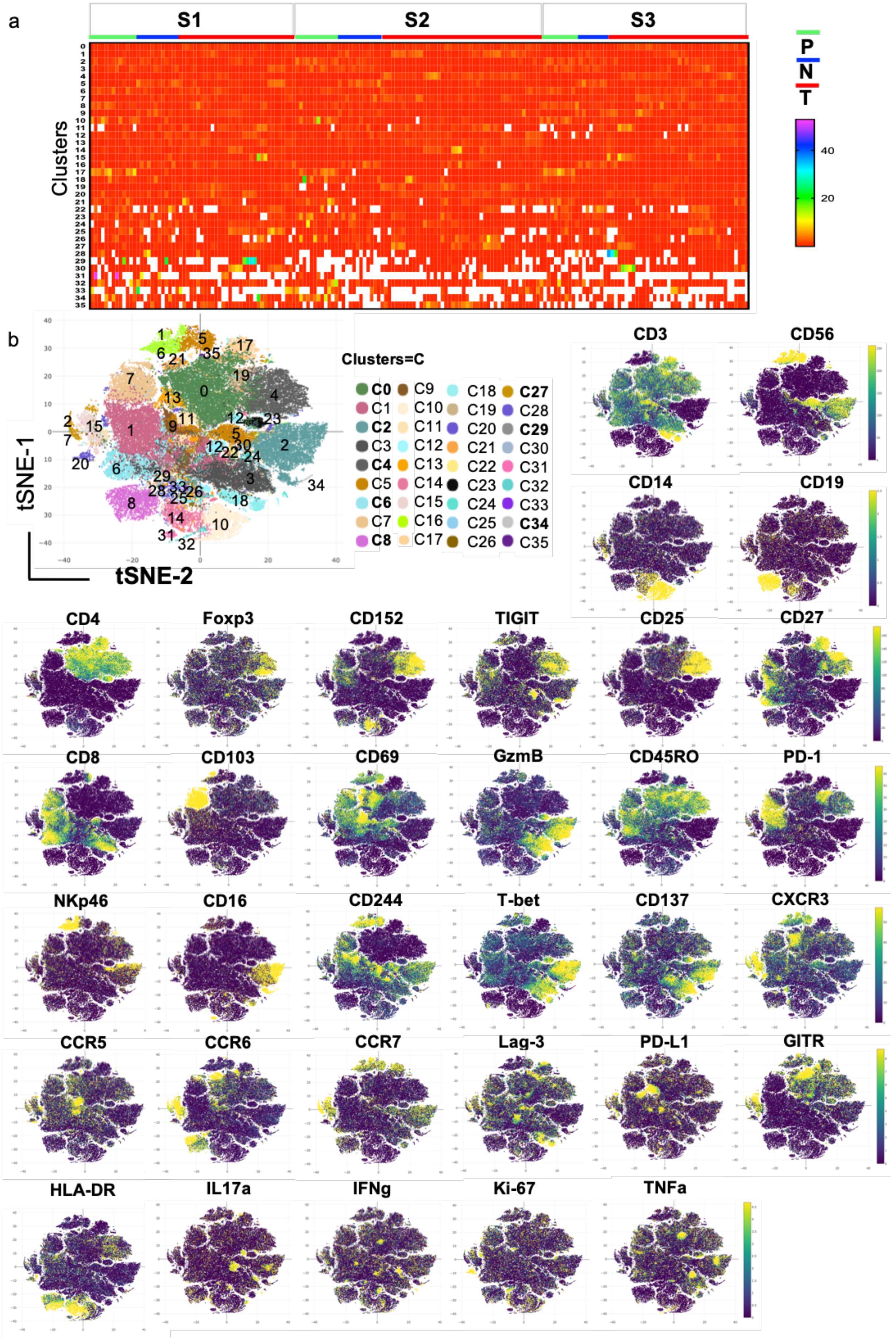

c

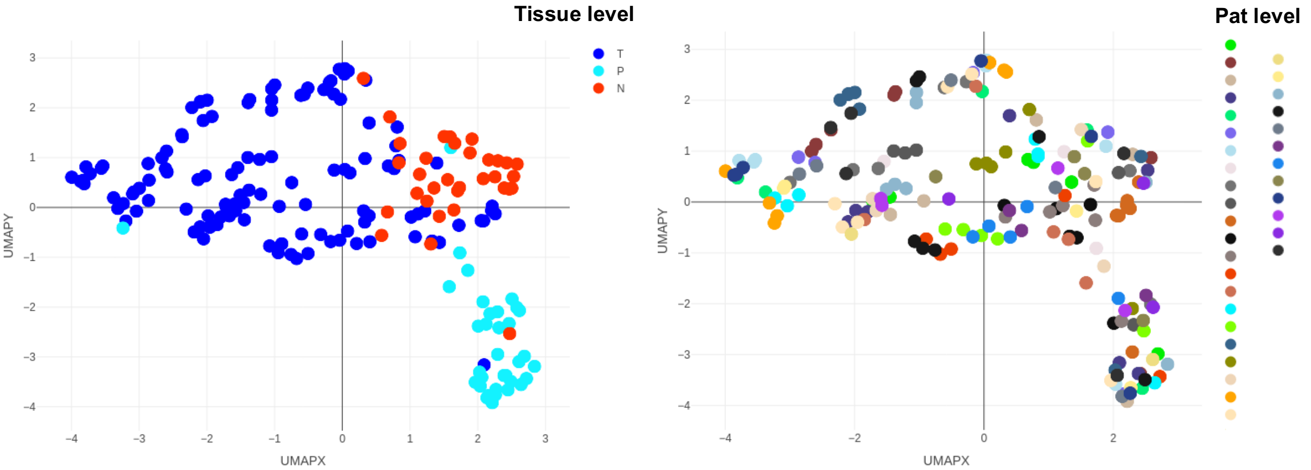

d

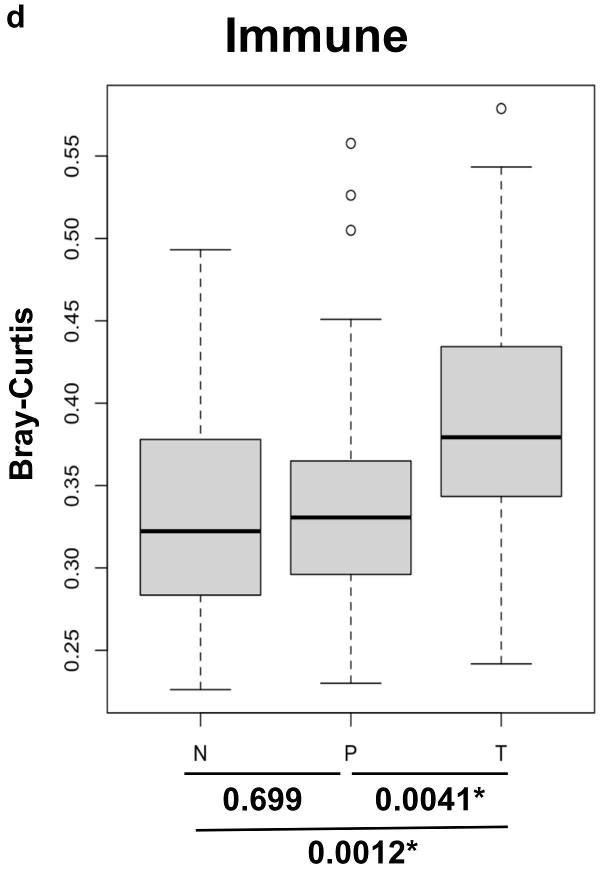

Supplementary Fig. 1: Increased diversity in immune clusters from HCC tumours.
a, Frequencies of immune clusters (y-axis) with each column represents one sample from P, N or T in Stage (S) 1, 2 or 3 HCC patients (each represented by a colour code).
b, tSNE plots showing relative marker expression for n=33 markers corresponded to the 36 immune clusters shown on top left plot.
c, Umap plot showing distributions of immune clusters according to tissue level: tumour (T) displays more heterogeneity as compared to adjacent non-tumour tissues (N) and peripheral blood (P) (left); or according to patient (Pat) level with each patient data point marked with colour (right): showing no distinct pat level distribution.
d, Immune diversity is measured as distance between each immune clusters according to tissues: N (n=33), P (n=34) or T (n=119). Adjusted two-sided p values as shown below; ** $p < 0.01$ by Tukey's HSD (honestly significant difference) test. Boxplots show median and the whiskers represent minimum and maximum values with the box edges showing the first and third quartiles. Data points beyond the whiskers are outliers and plotted as circles.

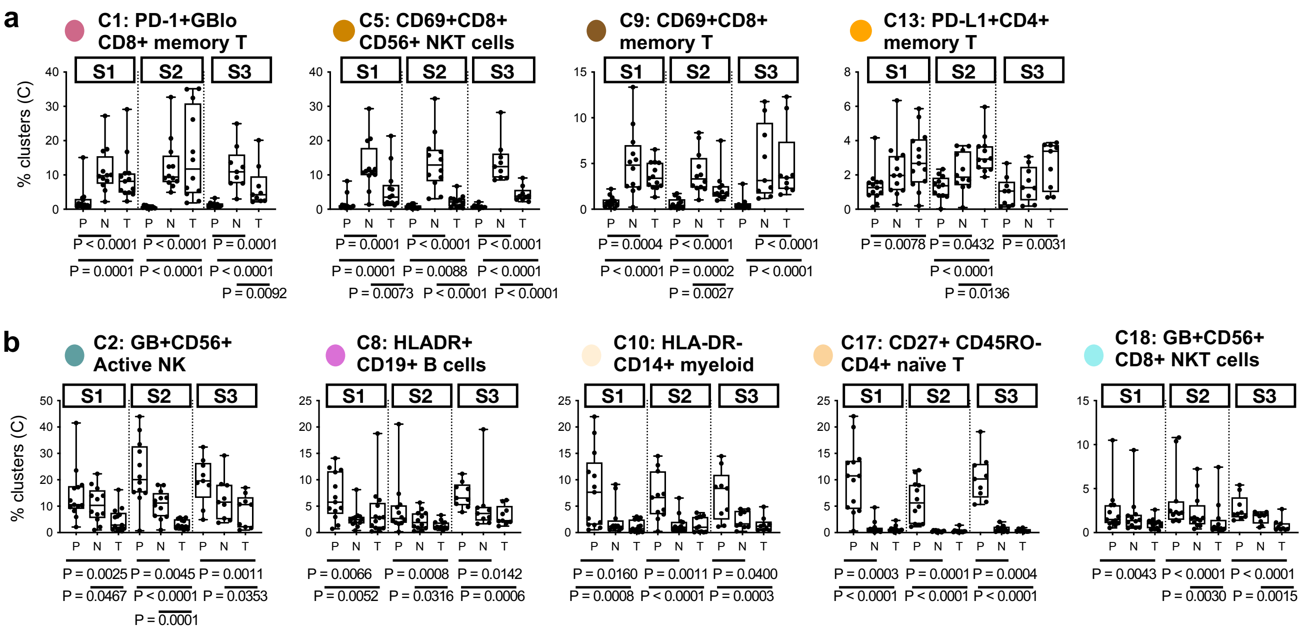

Supplementary Fig. 2: Immune clusters showing common differences across P, N and T in all three stages of HCC

a, Graphs showing percentages of C1, C5, C9 and C13 with significant enrichment in N and T versus P.
b, Graphs showing percentages of C2, C8, C10, C17 and C18 with significant enrichment in P versus N and T.
a, b, Clusters phenotypes are provided on top of each graph. Boxplots show median and the whiskers represent minimum and maximum values with the box edges showing the first and third quartiles. One-way ANOVA test with two-sided p values by unpaired Mann-Whitney U (MWU) tests for two-group comparisons. TNM stage I, II and III (S1, S2 and S3). n_{PS1}=13, n_{PS2}=12, n_{PS3}=9, n_{NS1}=12, n_{NS2}=12, n_{NS3}=9, n_{TS1}=13, n_{TS2}=12, n_{TS3}=9.

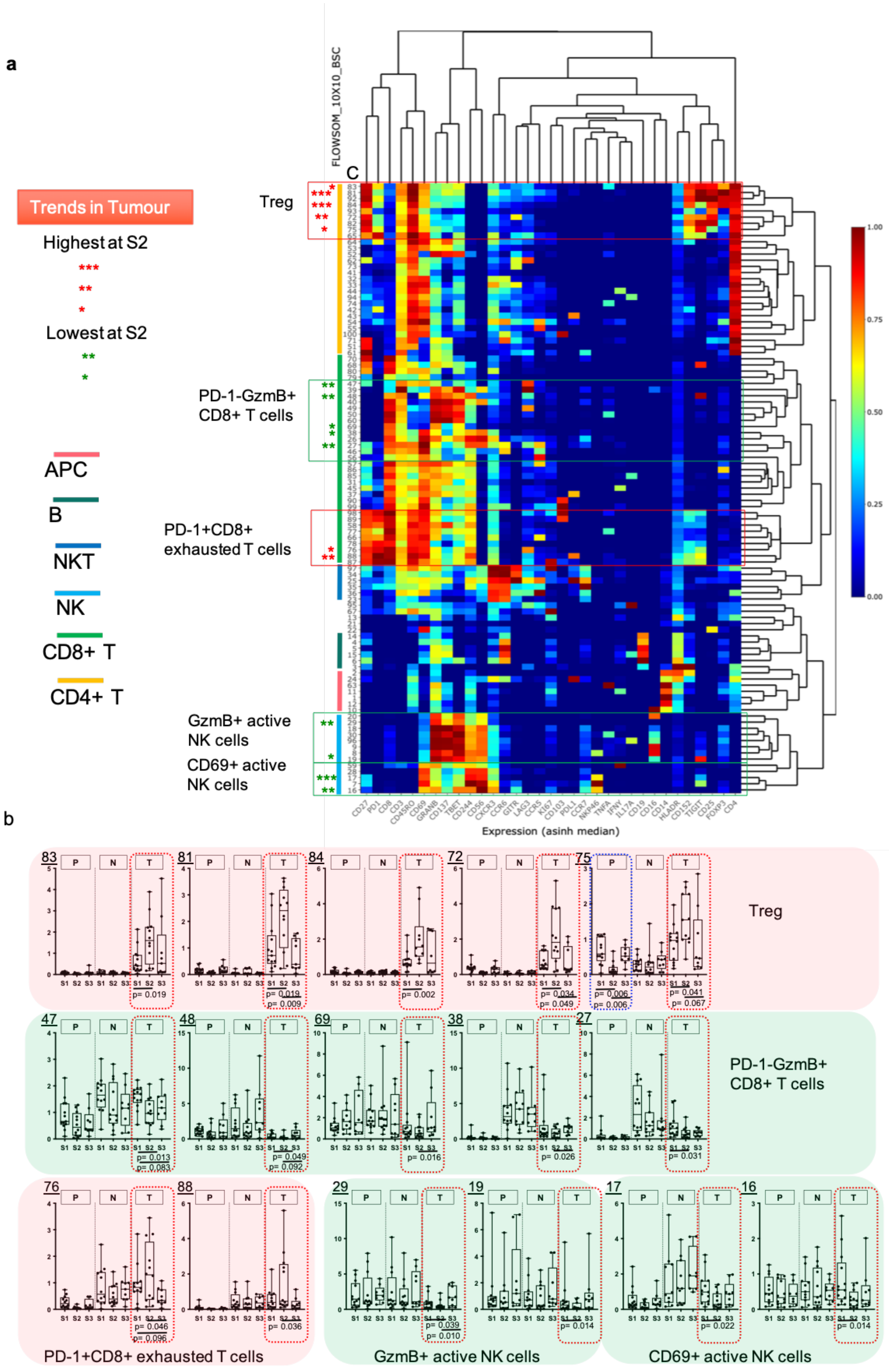
a
Trends in Tumour
Highest at S2
***
**
*
Lowest at S2
**
*
APC
B
NKT
NK
CD8+ T
CD4+ T
Treg
PD-1-GzmB+ CD8+ T cells
PD-1+CD8+ exhausted T cells
GzmB+ active NK cells
CD69+ active NK cells
FLOWSOM_10X10_BSC
C
CD27 PD1 CD8 CD3 CD45RO CD69 GRANB CD137 TBET CD244 CD56 CXCR3 CCR6 GITR LAG3 CCR5 KI67 CD103 PDL1 CCR7 NKP46 TNFA IFNY IL17A CD19 CD16 CD14 HLADR CD152 TIGIT CD25 FOXP3 CD4
Expression (asinh median)
1.00
0.75
0.50
0.25
0.00
b
P
N
T
S1 S2 S3
83 p= 0.019
81 p= 0.019 p= 0.009
84 p= 0.002
72 p= 0.034 p= 0.049
75 p= 0.006 p= 0.006 p= 0.041 p= 0.067
Treg
47 p= 0.013 p= 0.083
48 p= 0.049 p= 0.092
69 p= 0.016
38 p= 0.026
27 p= 0.031
PD-1-GzmB+ CD8+ T cells
76 p= 0.046 p= 0.096
88 p= 0.036
PD-1+CD8+ exhausted T cells
29 p= 0.039 p= 0.010
19 p= 0.014
GzmB+ active NK cells
17 p= 0.022
16 p= 0.014
CD69+ active NK cells

c

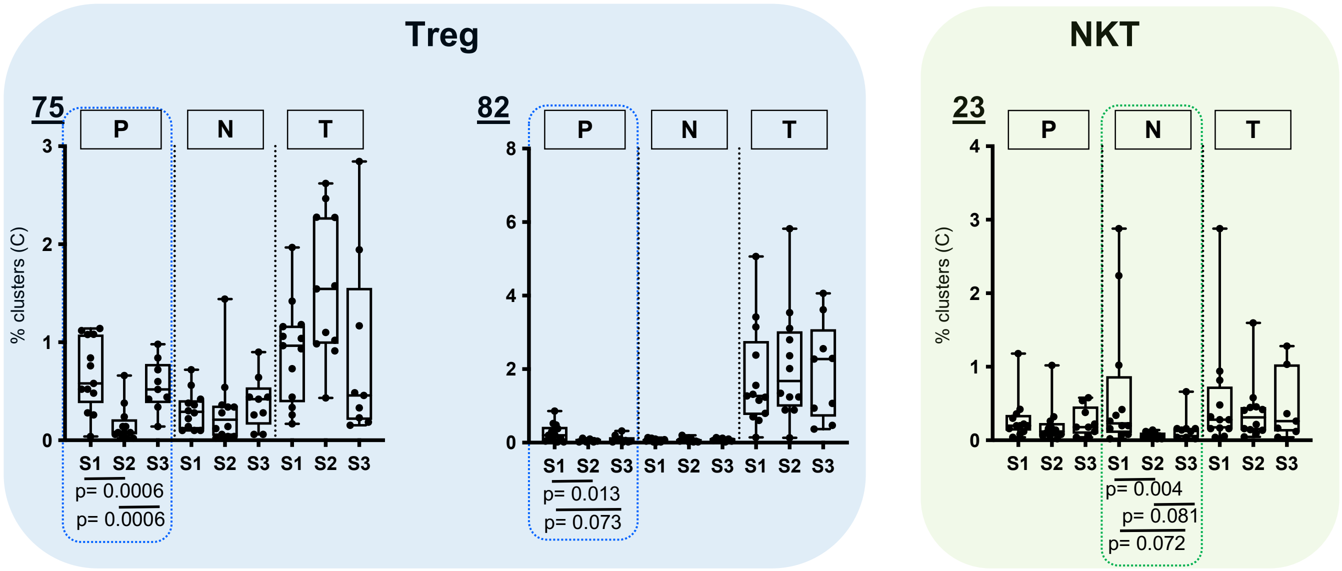

Supplementary Fig. 3: HCC immune landscapes analysis from different stages using FlowSom algorithm

a, Heatmap representation of the 10x10=100 FlowSom immune clusters (rows) with normalised protein expression of 33 markers (columns) from all samples. The colour bars and boxes indicate the major immune cell lineages and subsets. *, **, *** denote two-sided p values < 0.1, <0.05, <0.01 and <0.001 respectively by one-way ANOVA test with Tukey post-hoc multiple comparison test, marked with either red to show enrichment in S2 tumours or green as depletion in S2 tumours.

b, Graphs showing percentages of clusters with significant differences across TNM stages in tumour- T (red dashed line box). Background boxes in red show clusters enriched while in green show clusters depleted in S2 tumours. Trends in PBMC (P) are showed as blue dashed line box.

c, Graphs showing percentages of clusters with significant differences across TNM stages in PBMC-P (blue dashed line box) or NILs- N (green dashed line box).

b,c Boxplots show median and the whiskers represent minimum and maximum values with the box edges showing the first and third quartiles. One-way ANOVA test with two-sided p values by unpaired Mann-Whitney U (MWU) tests for two-group comparisons were indicated below respectively. TNM stage I, II and III (S1, S2 and S3). n_{PS1}=13, n_{PS2}=12, n_{PS3}=9, n_{NS1}=12, n_{NS2}=12, n_{NS3}=9, n_{TS1}=13, n_{TS2}=12, n_{TS3}=9.

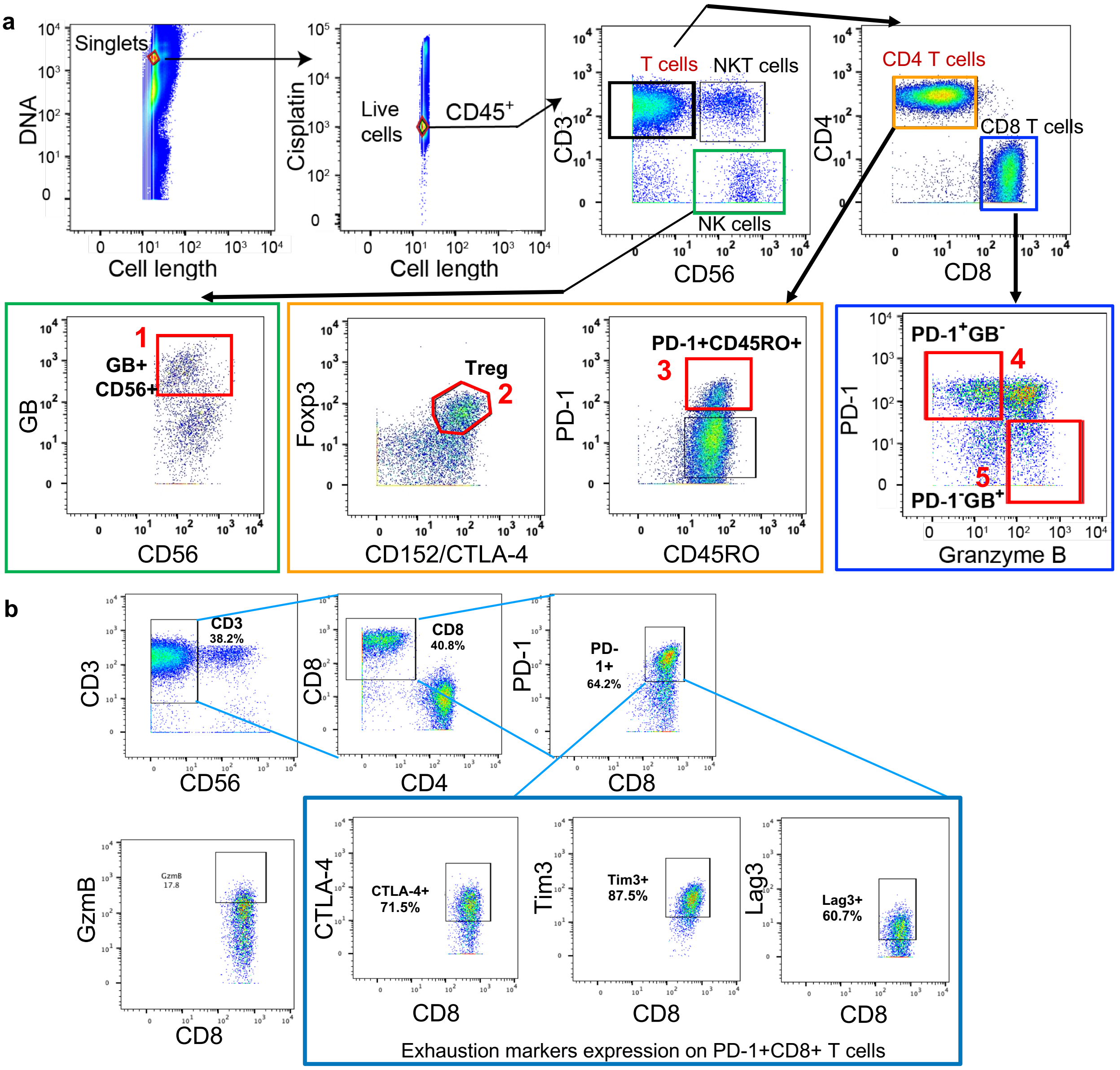

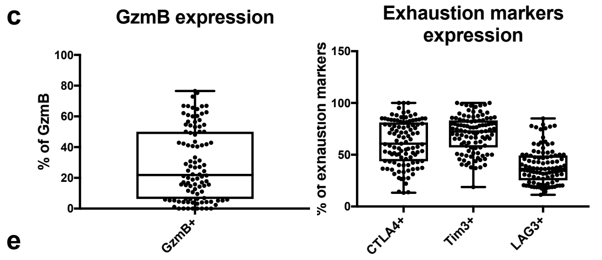

d

	GzmB	CTLA-4	Tim3	LAG3
**Median**	21.85	66.2	72.4	36.3
**Minimum**	0	14.3	18.8	10.4
**Maximum**	76.5	100	100	85.1
**Std. Deviation**	23.05	21.71	17.61	17.07

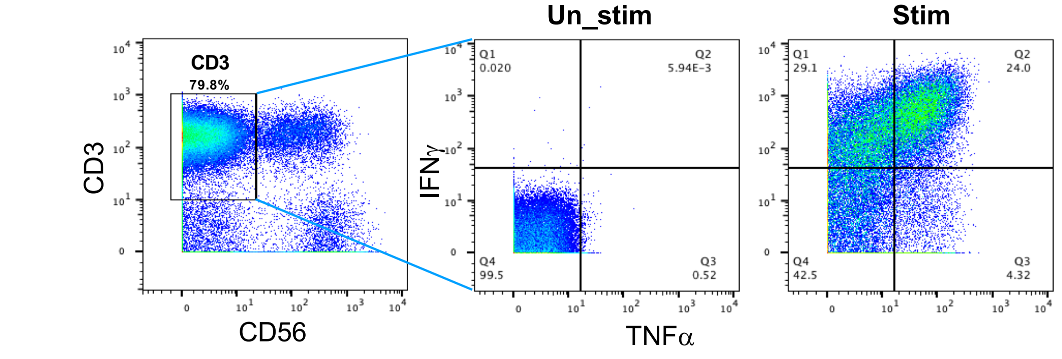

Supplementary Fig. 4: Manual gating for differentially expressed immune subsets in tumours from different TNM stages

a, Representative dot plots showing the manual gating of immune subsets using FlowJo. Singlets are gated out as cells positive for iridium DNA intercalator (DNA^+, y-axis) of a specific cell length (x-axis). All live immune cells are gated for Cisplatin(Live/dead dye)- $CD45^+$ populations. $CD3^+CD56^-$ T cells (black box) and CD3-CD56+ NK cells (green box) are gated. NK cells are further gated for: **1.** GB^+CD56^+ active NK cells. $CD3^+$ T cells are gated for $CD4^+$ (orange box) or $CD8^+$ (blue box) T cell subsets. $CD4^+$ T cells are then gated for **2.** $Foxp3^+$ $CTLA\text{-}4^+$ $CD4^+$ Treg and **3.** $PD\text{-}1^+CD45RO^+CD4^+$ memory T cells. While $CD8^+$ T cells are gated for **4.** $PD\text{-}1^+GB^-$ exhausted CD8+ T cells and **5.** $PD\text{-}1^-GB^+$ activated $CD8^+$ T cells. The gated immune subsets are marked with red boxes and numbered accordingly.

b, Representative dot plots showing the manual gating of exhausted $PD\text{-}1^+CD8^+$ T cells and the expression of cytokine, granzyme B (GzmB), other exhaustion markers (CTLA-4, Tim-3 and Lag3) using FlowJo.

c, Boxplots showing percentage of $GzmB^+$, $CTLA\text{-}4^+$, $Tim3^+$ and $Lag3^+$ populations from $PD\text{-}1^+$ exhausted CD8+ T cells in TILs (total tumour sectors= 136). Boxplots show median and the whiskers represent minimum and maximum values with the box edges showing the first and third quartiles.

d, Table showing median, maximum, minimum and standard (std) deviation of percentage of $GzmB^+$, $CTLA\text{-}4^+$, $Tim3^+$ and $Lag3^+$ populations from $PD\text{-}1^+$ exhausted $CD8^+$ T cells in TILs (total tumour sectors= 136).

e, Representative dot plots showing the manual gating of $TNFa^+IFNg^+$ active $CD3^+$ T cells using FlowJo.

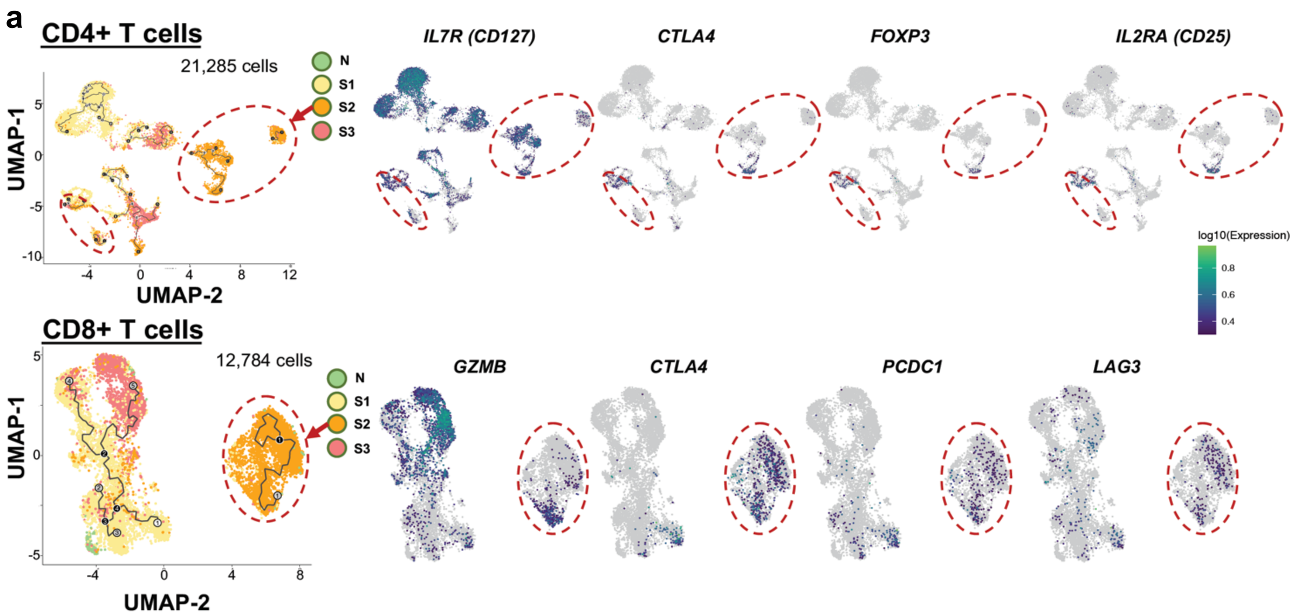

Supplementary Fig. 5: Single-cell RNA analysis of HCC-infiltrating immune cells from different TNM stages

a, Pseudotime trajectory ordering of tumour-infiltrating $CD4^+$ ($CD8^-CD3^+$) and $CD8^+$ ($CD8^+CD3^+$) T cells according to the starting point of S1 tumours, versus N, S2 tumours and S3 tumours. Expression level of S2-enriched genes are marked by red dotted circles and arrows.

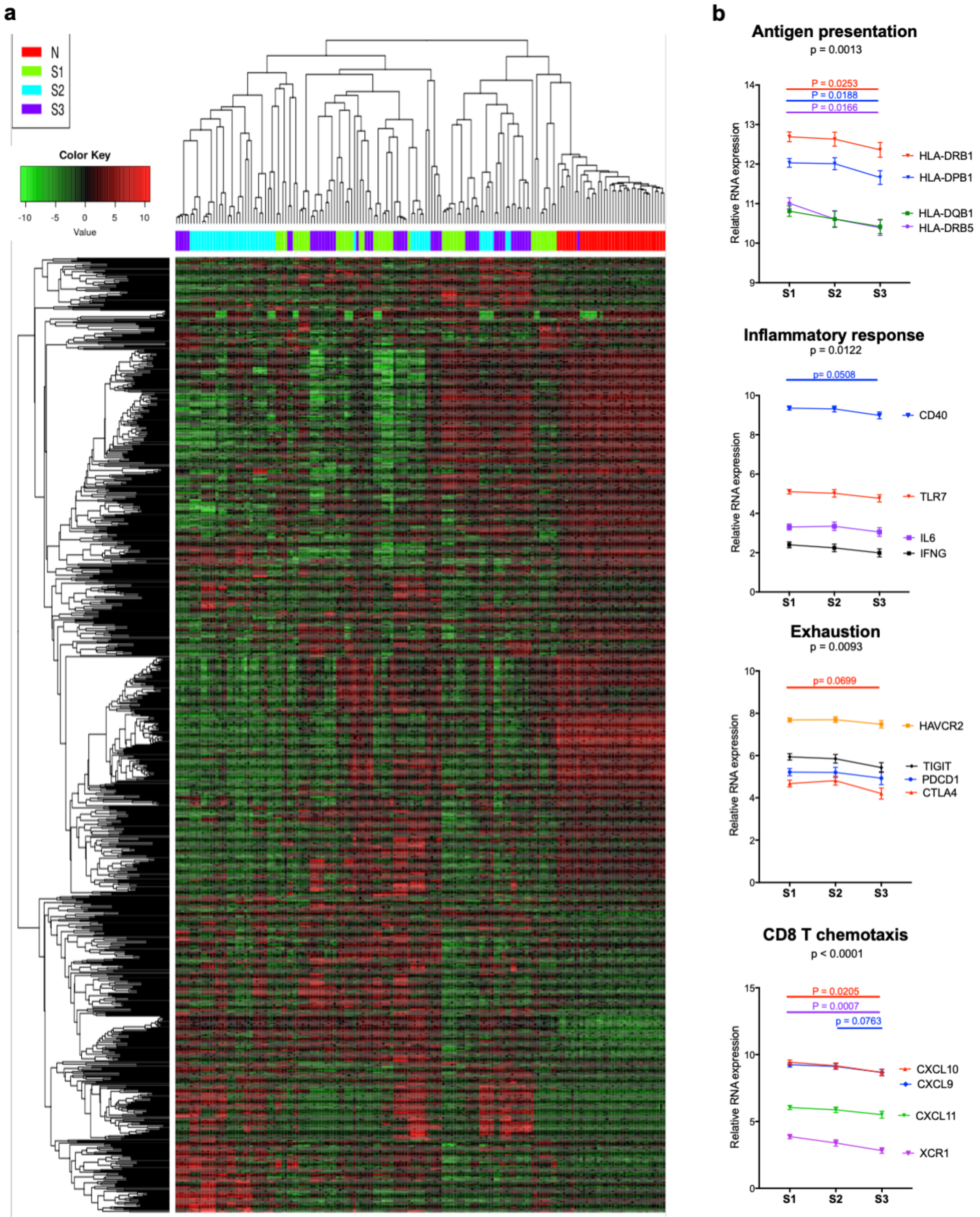

Supplementary Fig. 6: Differentially expressed genes (DEGs) among samples from different TNM stages
a, Heatmap showing DEGs from N, and S1-S3 HCC tumours, each denoted by the colour code.
b, Downregulation of multiple genes involved in key immune pathways across tumour stages in TCGA liver cancer cohort (S1, n=151; S2, n=75 and S3 n=71). Two-way Anova test followed by Two-sided p values calculated with respective unpaired Tukey's pairwise comparison test colour coded by each gene. Graphs show mean with standard error of the mean. n_N= 37, n_{S1}= 43, n_{S2}= 43 and n_{S3} = 45.

a

CTNNB1-mutated HCC

Over-expressed genes

Gene Symbol
NKD1
AXIN2
ROCK2
SALL1
TLE1
DVL2
CTNNBIP1
SMAD3
TCF7
BRD7
DAAM1
CUL1
PPP3CB
DLG1
RUVBL1
TBL1XR1
SENP2

Under-expressed genes

Gene Symbol
CTBP2
WNT4
TCF7L1
ARRB1
MAP1B
NFATC1
PLAU
WNT2
FSTL1
CXXC4
CDH1
FZD1
PRKCD
PLCB2
CSNK1E
PRKCB1
NKD2
WNT10A
FZD10

b

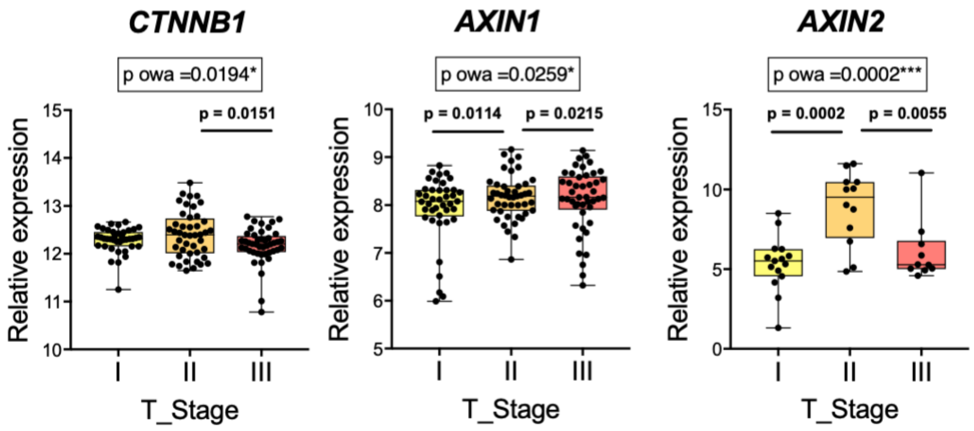

Supplementary Fig. 7: Differentially expressed genes in CTNNB1/Wnt signaling pathways in HCC tumours.
a, List of genes over-expressed or under-expressed in CTNNB1-mutated HCC tumours**.** adapted from *Lachenmayer et al Clin Can Res 2012*.
b, Expression of selected key genes involved in Wnt signaling pathways across tumour stages. Boxplots show median and the whiskers represent minimum and maximum values with the box edges showing the first and third quartiles. One-way ANOVA test with two-sided P values by Dunn's post-hoc multiple comparison test are as indicated. TNM stage I, II and III (S1, S2 and S3). n_{S1}= 43, n_{S2}= 43 and n_{S3} = 45

a

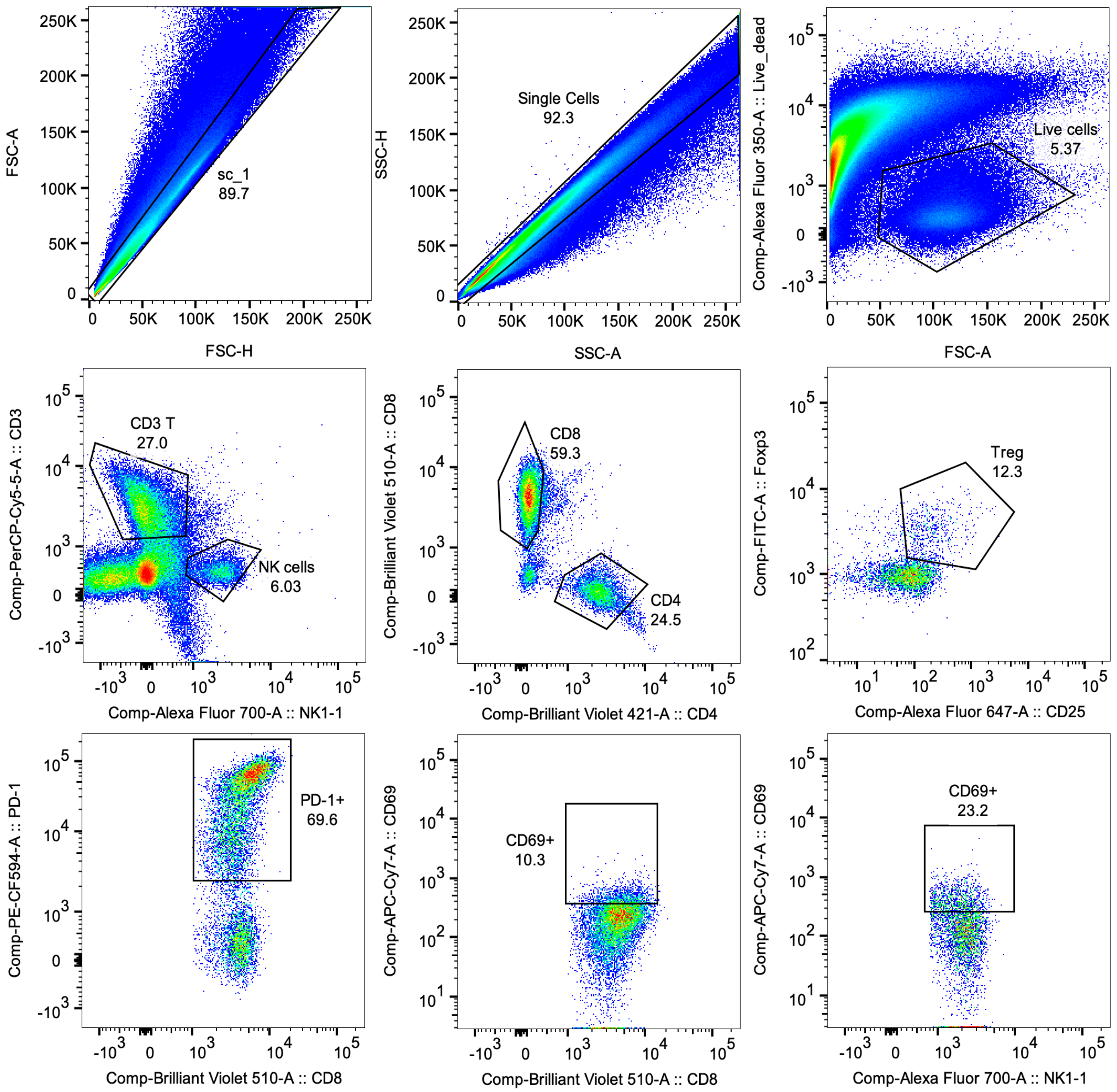

Supplementary Fig. 8: Gating strategy for immune cells isolated from murine HCC
a, Representative dot plots showing gating strategy for immune cells isolated from HCC models analyzed with flow cytometry.

Supplementary Tables:

Supplementary Table 1: Clinical and demographic information of the 38 HCC patients

Variables	Stage 1	Stage 2	Stage 3	P value
**n**	**16**	**12**	**10**	
**Age**<br>median (min, max)	66 (46, 76)	71.5 (47, 82)	65 (43, 78)	0.2443
**Gender**				
Male	12 (75.0%)	9 (75.0%)	10 (100.0%)	0.216
Female	4 (25.0%)	3 (25.0%)	0 (0.0%)	
**Race**				
Chinese	14 (87.5%)	11 (91.7%)	6 (60.0%)	0.1174
Others	2 (12.5%)	1 (8.3%)	4 (40.0%)	
**Grade**				
I&II	9 (56.3%)	7 (58.3%)	5 (50.0%)	0.9212
III&IV	7 (43.7%)	5 (41.7%)	5 (50.0%)	
**Viral status**				
Hep B	11 (68.8%)	8 (66.7%)	6 (60.0%)	0.8979
NV	5 (31.2%)	4 (33.3%)	4 (40.0%)	
**Tumour size [cm]**<br>median (min, max)	3.3 (1.8, 15)	6.85 (3, 14)	8.0 (5.5, 14)	0.0064**
**Total no. of sectors**	46	45	45	NA
**Tumour multiplicity**				
1	16 (100.0%)	11 (91.7%)	5 (50.0%)	0.0021**
>1	0 (0.0%)	1 (8.3%)	5 (50.0%)	
**AFP level (ng/ml)**<br>median (min, max)	8.0 (2.8, 8920)	23.7 (1.5, 54458)	103 (1.9, 60500)	0.4257
**MVI**				
Yes	0 (0.0%)	10 (83.3%)	6 (60.0%)	<0.0001****
No	16 (100.0%)	2 (16.7%)	4 (40.0%)	
**Fibrosis status**				
F0	3 (18.8%)	1 (8.3%)	0 (0.0%)	0.7884
F1	1 (6.3%)	2 (16.7%)	2 (20.0%)	
F2	4 (25.0%)	2 (16.7%)	1 (10.0%)	
F3	4 (25.0%)	3 (25.0%)	4 (40.0%)	
F4	4 (25.0%)	4 (33.3%)	3 (30.0%)	

Footnote:
Stage TNM: Version 8
Viral Status: Hep B= Hepatitis B; NV = non-viral-related HCC (patients with no detectable HBV surface/core antigen)
AFP: Alpha-fetoprotein
MVI: microvascular invasion
Numerical features of the three HCC stages were compared with the One-way ANOVA test, categorical features using the Chi-square test . ** p < 0.01 and **** p < 0.0001

Supplementary Table 2: Antibodies used for CyTOF staining.

Antibodies	Clone	Vendor	Catalog number	Metal Isotopes	Working Concentration
**CD45 (Barcode 1)**	HI30	Fluidigm	#3089003B	89	5ug/ml
**CD14**	TüK4	Lifetechnologies	#Q10064	112/114	5ug/ml
**CD45 (Barcode 2)**	HI30	Biolegend	#304002	115	5ug/ml
**HLA-DR**	L243	Biolegend	#307602	139	6ug/ml
**CD19**	HIB19	Biolegend	#302202	141	5ug/ml
**CD45RO**	UCHL1	Biolegend	#304202	142	5ug/ml
**CD3**	UCHT1	Biolegend	#300402	143	5ug/ml
**CD8**	SK1	Biolegend	#344702	144	5ug/ml
**T-bet**	4B10	Biolegend	#644802	145	5ug/ml
**TNFα**	Mab11	Biolegend	#502902	146	3ug/ml
**PD-1**	EH12.2H7	Biolegend	#329902	147	7ug/ml
**CD4**	SK3	Biolegend	#344602	148	4ug/ml
**CD103**	B-Ly7	Ebioscience	#14-1038-82	150	6ug/ml
**TIGIT**	MBSA43	Ebioscience	#16-9500-85	151	6ug/ml
**NKp46**	9.00E+02	Biolegend	#331902	152	5ug/ml
**CD25**	2A3	BD bioscience	#347640	153	5ug/ml
**CD27**	O323	Biolegend	#302802	154	5ug/ml
**CD152**	BNI3	BD bioscience	#555850	155	5ug/ml
**PD-L1**	29E.2A3	Biolegend	#329719	156	5ug/ml
**CD244**	C1.7	Biolegend	#329502	157	6ug/ml
**LAG-3**	17B4	Abcam	#ab40466	159	5ug/ml
**CCR7**	G043H7	Biolegend	#353202	161	7ug/ml
**CD56**	NCAM16.2	BD bioscience	#559043	162	4ug/ml
**CXCR3**	G025H7	Biolegend	#353702	163	5ug/ml
**GITR**	621	Biolegend	#311602	164	6ug/ml
**FoxP3**	PCH101	Ebioscience	#14-4776-82	165	3ug/ml
**Ki67**	20Raj1	Ebioscience	#14-5699-82	166	3ug/ml
**IFN-γ**	B27	Biolegend	#506502	168	4ug/ml
**IL-17A**	BL168	Biolegend	#512302	169	3ug/ml
**CCR6**	G034E3	Biolegend	#353402	170	6ug/ml
**CD45 (Barcode 3)**	HI30	Biolegend	#304002	172	5ug/ml
**GranzymeB**	CLB-GB11	Abcam	#ab103159	173	2ug/ml
**CD137**	4B4-1	Biolegend	#309802	174	4ug/ml
**CCR5**	T21/8	Biolegend	#321402	175	7ug/ml
**CD69**	FN50	Biolegend	#310902	176	5ug/ml
	Ir Intercalator	Fluidigm	#201192B	191/193	0.25uM
**CD16**	3G8	Fluidigm	#3209002B	209	5ug/ml

Supplementary Table 3: List of 13 (out of 36) Phenograph clusters showing significant differences in cell fractions (%CD45+) between tumour stages S1, S2, S3 in tissues P, N and T. +, *, **, *** denote p< 0.1, <0.05, <0.01 and <0.001, respectively for one-way ANOVA multi-group and Mann-Whitney U (MWU) two-group comparisons.

Stage 1

Cluster	P ANOVA	P MWU P vs N	P MWU P vs T	P MWU N vs T	Median P	Median N	Median T
0	0.0024**	0.7283	0.0107*	0.0020**	6.46	5.30	10.92
1	0.0025**	0.0001***	0.0000***	0.2261	1.32	9.77	7.86
2	0.0021**	0.4059	0.0025**	0.0467*	10.56	10.30	3.64
4	0.0000***	0.0135*	0.0000***	0.0000***	1.86	0.88	6.34
5	0.0003***	0.0001***	0.0001***	0.0073**	0.92	10.99	3.82
7	0.0001***	0.0015**	0.0000***	0.7777	0.40	3.46	4.40
8	0.0061**	0.0066**	0.0052**	0.7679	5.76	2.44	2.00
9	0.0000***	0.0004***	0.0000***	0.164	0.70	4.82	2.96
10	0.0000***	0.0160*	0.0008***	0.2985	7.64	1.19	0.90
13	0.0346*	0.0868+	0.0078**	0.3422	1.26	1.97	2.76
17	0.0000***	0.0003***	0.0000***	0.5549	10.75	0.50	0.40
18	0.0201*	0.4371	0.0043**	0.0703+	1.48	1.37	0.94
19	0.0000***	0.0682+	0.0000***	0.0004***	0.20	0.78	2.26

Stage 2

Cluster	P ANOVA	P MWU P vs N	P MWU P vs T	P MWU N vs T	Median P	Median N	Median T
0	0.0000***	0.7987	0.0000***	0.0000***	6.48	6.29	14.63
1	0.0002***	0.0000***	0.0000***	0.6177	0.47	9.39	13.84
2	0.0000***	0.0045**	0.0000***	0.0001***	20.09	11.19	1.47
4	0.0000***	0.3121	0.0000***	0.0000***	0.51	0.29	11.83
5	0.0000***	0.0000***	0.0088**	0.0000***	0.56	12.90	1.55
7	0.0005***	0.0000***	0.0000***	0.2781	0.33	5.25	4.50
8	0.0015**	0.2189	0.0008***	0.0316*	2.66	1.91	0.70
9	0.0053**	0.0000***	0.0002***	0.0027**	0.41	3.31	1.61
10	0.0001***	0.0011**	0.0000***	0.2324	6.88	1.02	0.74
13	0.0000***	0.0432*	0.0000***	0.0136*	1.37	1.87	3.03
17	0.0000***	0.0001***	0.0000***	0.7726	5.62	0.22	0.18
18	0.0145*	0.0780+	0.0000***	0.0030**	2.29	1.56	0.55
19	0.0000***	0.0044**	0.0000***	0.0000***	0.06	0.36	2.93

Stage 3

Cluster	P ANOVA	P MWU P vs N	P MWU P vs T	P MWU N vs T	Median P	Median N	Median T
0	0.0003***	0.0770	0.0075**	0.0003***	8.28	5.36	16.69
1	0.0028**	0.0001***	0.0000***	0.0092**	1.20	10.93	3.71
2	0.0022**	0.0770+	0.0011**	0.0353*	19.60	11.52	3.81
4	0.0001***	0.0770+	0.0002***	0.0000***	1.80	0.78	8.90
5	0.0000***	0.0000***	0.0000***	0.0000***	0.58	12.42	3.19
7	0.0000***	0.0004***	0.0000***	0.0482*	0.32	2.66	6.54
8	0.0193*	0.0142*	0.0006***	0.1276	6.54	3.62	2.40
9	0.0189*	0.0005***	0.0000***	0.8973	0.40	3.14	3.05
10	0.0000***	0.0400*	0.0003***	0.0630	8.38	1.64	0.83
13	0.0045**	0.2508	0.0031**	0.0649+	1.06	1.26	2.68
17	0.0000***	0.0004***	0.0000***	0.0726+	10.16	0.56	0.26
18	0.0000***	0.1903	0.0001***	0.0015**	2.13	1.96	0.51
19	0.0002***	0.0315*	0.0001***	0.0208*	0.30	0.70	1.51

Supplementary table 4: Phenograph Clusters (out of n=36) showing significant differences in cell fractions (%CD45+) between tumour stages S1, S2, S3 in tissues P, N and T. +, *, **, *** denote p< 0.1, <0.05, <0.01 and <0.001, respectively for one-way ANOVA multi-group and Mann-Whitney U (MWU) two-group comparisons.

Peripheral blood (P)

Cluster	P ANOVA	p MWU S1 vs S2	p MWU S1 vs S3	P MWU S2 vs S3	Median S1	Median S2	Median S3
**4**	0.0112*	0.0160*	0.5123	0.0184*	1.86	0.51	1.80
**17**	0.0726+	0.0868+	1.0000	0.0409*	10.75	5.62	10.16
**20**	0.0802+	0.0298*	0.5256	0.0879+	1.48	1.08	1.36

Non-tumour liver (N)

Cluster	P ANOVA	p MWU S1 vs S2	p MWU S1 vs S3	P MWU S2 vs S3	Median S1	Median S2	Median S3
**7**	0.0532+	0.1005	0.3554	0.0056**	3.46	5.25	2.66
**15**	0.0751+	0.0404*	0.3100	0.3026	0.99	0.58	0.76

Tumour (T)

Cluster	P ANOVA	p MWU S1 vs S2	p MWU S1 vs S3	P MWU S2 vs S3	Median S1	Median S2	Median S3
**6**	0.0289*	0.0220*	0.0514^{+}	0.6511	6.59	2.42	3.77
**19**	0.0362*	0.3203	0.0455*	0.0073**	3.15	3.24	1.46
**4**	0.0392*	0.0188*	0.7438	0.1694	7.03	13.48	8.72
**2**	0.0501+	0.4696	0.1858	0.0471*	2.85	2.57	10.62
**1**	0.0798+	0.3760	0.1264	0.0458*	8.14	11.73	4.23
**9**	0.0846+	0.0096**	0.9479	0.0184*	3.38	1.73	3.45

Supplementary table 5: FlowSOM clusters (out of n=100) showing significant differences in cell fractions (%CD45+) between tumour stages S1, S2, S3 in tissues P, N and T. +, *, **, *** denote p< 0.1, <0.05, <0.01 and <0.001, respectively for one-way ANOVA multi-group and Mann-Whitney U (MWU) two-group comparisons.

Peripheral blood (P)

Cluster	P ANOVA	p MWU S1 vs S2	p MWU S1 vs S3	P MWU S2 vs S3	Median S1	Median S2	Median S3
**75**	0.0180*	0.0006***	0.0006***	0.403	0.58	0.08	0.52
**82**	0.0530+	0.013*	0.586	0.073+	0.20	0.01	0.06

Non-tumour liver (N)

Cluster	P ANOVA	p MWU S1 vs S2	p MWU S1 vs S3	P MWU S2 vs S3	Median S1	Median S2	Median S3
**23**	0.0520+	0.004**	0.081+	0.072+	0.23	0.05	0.14

Tumour (T)

Cluster	P ANOVA	p MWU S1 vs S2	p MWU S1 vs S3	P MWU S2 vs S3	Median S1	Median S2	Median S3
**81**	0.0003***	0.029*	0.009**	0.431	0.60	2.16	0.65
**84**	0.0004***	0.002**	0.237	0.916	0.62	1.76	1.20
**17**	0.0012**	0.022*	0.193	0.431	0.98	0.18	0.42
**72**	0.0015**	0.034*	0.049*	0.269	0.54	1.75	0.36
**88**	0.0024**	0.224	0.036*	0.310	0.34	0.50	0.15
**75**	0.0033**	0.041*	0.067+	0.471	0.64	1.46	0.43
**16**	0.0035**	0.014*	0.452	0.209	0.58	0.14	0.22
**29**	0.0039**	0.039*	0.01*	0.071+	0.68	0.24	0.69
**47**	0.0070**	0.013*	0.475	0.083+	1.52	0.75	1.10
**48**	0.0071**	0.049*	0.092+	0.471	0.22	0.07	0.27
**27**	0.0080**	0.031*	0.422	0.169	0.78	0.22	0.48
**19**	0.0110*	0.014*	0.560	0.213	0.26	0.04	0.16
**69**	0.0250*	0.574	0.016*	0.324	0.72	0.44	0.88
**83**	0.0460*	0.019*	0.129	>0.999	0.34	1.40	0.51
**38**	0.0480*	0.314	0.026*	0.284	0.96	0.46	1.17
**76**	0.0480*	0.551	0.046*	0.096+	0.84	1.04	0.30

Supplementary Table 6: Up-regulated gene pathways in HCC tumours from different Stages (Immune GO ontology marked as **bold).** P values of Fisher's exact test were adjusted using the Benjamini-Hochberg method. FDR= false discovery rate.

1072 genes exclusively upregulated in "S1":

Term	Count	Genes	Fold Enrichment	P value adjusted	FDR
**GO:0006935~chemotaxis**	34	CXCL6, CXCL9, CCL11, C5AR2, C5AR1, PTAFR, FPR1, FPR2, CXCL14, CXCL2, CYR61, CXCL16, TYMP, SPN, CYSLTR1, CCL8, PLAU, CXCR3, CCRL2, CCL3, S1PR1, CCL2, CCR7, CCL18, CMKLR1, CCL23, XCR1, CCL22, CMTM3, CMTM6, FOSL1, CXCL10, CXCL11, HRG	4.815	5.00E-11	4.91E-11
**GO:0071356~cellular response to tumor necrosis factor**	32	CCL11, GATA3, THBS1, YBX3, IL18BP, CRHBP, ZFP36, CCL8, ADAMTS13, HYAL2, CCL4, ZC3H12A, CCL3, CCL2, HAS2, CCL19, CCL18, PCK1, CAMP, MAP3K5, POSTN, CCL23, EDN1, CCL22, VCAM1, KLF2, COL1A1, SFRP1, IL6, FABP4, XCL2, HAMP	5.026	6.54E-11	6.43E-11
**GO:0071347~cellular response to interleukin-1**	22	CCL23, EDN1, PTGIS, CCL11, CCL22, NFKB1, KLF2, IL6, SFRP1, CCL8, MYC, HYAL2, CCL4, ZC3H12A, CCL3, XCL2, CCL2, HAS2, CCL19, PCK1, CCL18, CAMP	5.353	1.31E-07	1.29E-07
**GO:0071346~cellular response to interferon-gamma**	18	GBP5, CCL23, CIITA, EDN1, CCL11, CCL22, NOS2, CCL8, ADAMTS13, MYC, CCL4, CCL3, XCL2, CCL2, CCL19, CCL18, TLR3, HLA-DPA1	5.455	4.47E-06	4.40E-06
**GO:0045087~innate immune response**	53	CD84, NRROS, CLEC4M, CLEC10A, IL1RAP, ADARB1, PRDM1, PIK3CG, DHX58, CASP4, B2M, CAMP, MAP3K5, ZBP1, ZNF683, TICAM2, RIPK2, MATK, TMEM173, CLEC4A, FGR, IL23A, TRIM14, CD300E, TLR10, TLR7, CLEC4E, TLR4, S100A8, TLR3, C1QB, CSF1R, C1QA, NLRX1, NLRC5, GATA3, RNF135, SEC14L1, IFI16, NLRP3, FYN, CD14, APOBEC3G, APOBEC3H, MX1, SH2D1B, DEFB132, NFKB1, NFKB2, AXL, SARM1, KLRD1, C1QC	2.129	7.45E-05	7.33E-05
**GO:0030593~neutrophil chemotaxis**	17	CCL23, EDN1, CSF3R, CCL11, CCL22, C5AR1, PPBP, CXCL3, PIK3CG, CCL8, CCL4, CCL3, PDE4B, XCL2, CCL2, CCL18, S100A8	4.450	1.51E-04	1.48E-04
GO:0043950~positive regulation of cAMP-mediated signaling	8	CXCL10, CXCL11, CXCL9, PTGIR, CXCR3, GNAS, RAPGEF2, PF4	11.517	2.20E-04	2.17E-04
GO:0007204~positive regulation of cytosolic calcium ion concentration	24	PTGFR, CD52, PTGIR, EDN1, XCR1, C5AR2, C5AR1, FPR1, FPR3, CACNA1C, PTH1R, FPR2, PIK3CG, CYSLTR1, C1QTNF1, DLG4, CXCR3, P2RY1, NPTN, BDKRB2, AGTR1, CCR7, S1PR4, TRPM4	3.094	3.79E-04	3.72E-04
GO:0007169~transmembrane receptor protein tyrosine kinase signaling pathway	18	NTRK2, CSF1R, FLT3, MATK, GFRA2, FGR, CD4, CD8B, KIT, NTF3, ERBB2, RAPGEF1, NPTN, BDKRB2, LCP2, FYN, ROR2, ANGPTL1	3.239	4.16E-03	4.09E-03
**GO:0002548~monocyte chemotaxis**	11	CCL23, IL6, CCL8, CCL11, CCL22, CCL4, CCL3, XCL2, CCL2, CCL19, CCL18	4.525	1.15E-02	1.13E-02
GO:0015671~oxygen transport	7	HBM, HBG2, MYC, HBB, HBA2, HBD, HBA1	8.062	1.18E-02	1.16E-02
**GO:0048247~lymphocyte chemotaxis**	9	CCL23, CCL8, CCL11, CCL22, CCL3, XCL2, CCL2, CCL19, CCL18	5.553	1.28E-02	1.26E-02
GO:0030816~positive regulation of cAMP metabolic process	5	CXCL10, CXCL11, CXCL9, CXCR3, PF4	14.396	1.38E-02	1.36E-02

GO:0051056~regulation of small GTPase mediated signal transduction	20	PLEKHG2, ARHGEF17, ARAP2, ARHGAP29, RHOF, SIPA1L2, ARHGAP15, RHOD, ARHGAP23, FGD4, AKAP13, SYDE1, ABR, ARHGAP20, ARHGAP31, OBSCN, ARHGAP30, DLC1, ARHGDIB, ARHGEF6	2.578	2.03E-02	2.00E-02
**GO:0002690~positive regulation of leukocyte chemotaxis**	7	CXCL6, CXCL10, IL6, CXCL11, CXCL9, PPBP, PF4	6.718	2.74E-02	2.70E-02
**GO:0034128~negative regulation of MyD88-independent toll-like receptor signaling pathway**	5	TICAM2, SARM1, CD14, TLR4, TLR3	10.797	4.46E-02	4.38E-02

867 genes exclusively upregulated in "S3":

Term	Count	Genes	Fold Enrichment	P value adjusted	FDR
GO:0006614~SRP-dependent cotranslational protein targeting to membrane	16	RPL3, RPS7, RPL31, RPLP1, RPL12, RPL9, RPL7A, RPS28, RPS16, RPS29, RPL37A, RPS3, RPL36, RPS20, RPL38, RPS27A	3.88	3.38E-02	3.37E-02
GO:0019083~viral transcription	17	RPL3, RPS7, RPL31, RPLP1, RPL12, RPL9, RPL7A, RPS28, RPS16, RPS29, RPL37A, RPS3, RPL36, RPS20, RPL38, RPS27A, NUPL2	3.46	3.67E-02	3.67E-02

85 common upregulated genes in "S1" and "S2":

Term	Count	Genes	Fold Enrichment	P value adjusted	FDR
GO:0005759~mitochondrial matrix	8	PDHX, LARS2, PCCA, FECH, PDHB, ABCE1, TRIT1, ACAT1	5.57	3.66E-02	3.66E-02

912 common upregulated genes in "S1" and "S3":

Term	Count	Genes	Fold Enrichment	P value adjusted	FDR
GO:0030574~collagen catabolic process	14	COL18A1, COL15A1, COL12A1, FURIN, COL3A1, MMP14, ADAMTS2, ADAMTS3, COL4A2, COL5A1, COL4A1, CTSL, COL6A3, COL8A1	4.50	1.82E-03	1.78E-03
GO:0003158~endothelium development	5	GJA1, GJA5, GJA4, KDR, CD34	17.15	8.84E-03	8.64E-03
GO:0046069~cGMP catabolic process	5	PDE10A, PDE1A, PDE2A, PDE5A, PDE9A	14.70	1.65E-02	1.61E-02
GO:0050919~negative chemotaxis	9	SEMA6B, SEMA5A, SEMA6A, SEMA6D, SEMA4C, PDGFA, APOA1, SLIT3, EFNA5	5.45	1.65E-02	1.61E-02
GO:0046777~protein autophosphorylation	21	PDGFRB, PDGFRA, EPHA4, FLT1, DAPK1, FLT4, INSR, PTK6, THY1, BMX, IGF1R, BCR, FES, CAMK4, KDR, PRKD2, SIK1, TEK, EPHA1, EPHB1, FGFR2	2.51	2.16E-02	2.11E-02
GO:0010544~negative regulation of platelet activation	5	THBD, PDGFRA, NOS3, PDGFB, PDGFA	12.86	2.59E-02	2.53E-02
GO:0007169~transmembrane receptor protein tyrosine kinase signaling pathway	14	BLK, LTK, FLT1, FLT4, INSR, PTK6, NGF, BMX, IGF1R, DOK5, KDR, CSPG4, TEK, PAG1	3.00	4.46E-02	4.35E-02

301 common upregulated genes in "S2" and "S3":

Term	Count	Genes	Fold Enrichment	P value adjusted	FDR
GO:0032200~telomere organization	7	HIST1H3A, HIST1H4H, HIST1H3B, HIST1H4C, HIST1H3C, HIST1H4D, HIST1H3E	17.01	2.02E-03	2.01E-03
GO:0051290~protein heterotetramerization	8	NLGN1, HIST1H3A, HIST1H4H, HIST1H3B, HIST1H4C, HIST1H3C, HIST1H4D, HIST1H3E	12.49	2.02E-03	2.01E-03
GO:0006335~DNA replication-dependent nucleosome assembly	7	HIST1H3A, HIST1H4H, HIST1H3B, HIST1H4C, HIST1H3C, HIST1H4D, HIST1H3E	14.35	3.37E-03	3.36E-03
GO:0045814~negative regulation of gene expression, epigenetic	8	HIST1H3A, PHF1, HIST1H4H, HIST1H3B, HIST1H4C, HIST1H3C, HIST1H4D, HIST1H3E	10.50	3.37E-03	3.36E-03
GO:0000183~chromatin silencing at rDNA	7	HIST1H3A, HIST1H4H, HIST1H3B, HIST1H4C, HIST1H3C, HIST1H4D, HIST1H3E	12.41	4.97E-03	4.96E-03
GO:0045815~positive regulation of gene expression, epigenetic	8	HIST1H3A, POLR1D, HIST1H4H, HIST1H3B, HIST1H4C, HIST1H3C, HIST1H4D, HIST1H3E	8.46	9.46E-03	9.44E-03

Supplementary Table 7: Down-regulated gene pathways in HCC tumours from different Stages. P values of Fisher's exact test were adjusted using the Benjamini-Hochberg method. FDR= false discovery rate.

1124 genes exclusively downregulated in "S1":

Term	Count	Genes	Fold Enrichment	P value adjusted	FDR
GO:0006334~nucleosome assembly	29	HIST1H2BN, HIST1H2BM, HIST1H2BO, HIST1H2BJ, HIST1H2BL, HIST1H2BK, CENPA, HIST1H3A, HIST1H1D, HIST1H1E, HIST1H3H, HIST1H3B, HIST1H1A, HIST1H3C, HIST1H1B, HIST1H3D, HIST1H3E, BRD2, HIST2H2BF, HIST1H4A, HIST1H2BF, HIST1H2BE, HIST1H4H, HIST1H2BG, HIST1H4C, HIST2H3D, HIST1H4D, HIST1H2BD, HIST1H4E	4.16	5.21E-07	5.19E-07
GO:0045814~negative regulation of gene expression, epigenetic	16	PHF1, BMI1, HIST1H4A, HIST1H3A, DNMT3B, HIST1H4H, HIST1H3H, TRIM27, HIST1H3B, HIST1H4C, HIST2H3D, HIST1H3C, HIST1H4D, HIST1H4E, HIST1H3D, HIST1H3E	5.47	1.48E-04	1.48E-04
GO:0006335~DNA replication-dependent nucleosome assembly	12	HIST1H4A, CHAF1A, HIST1H3A, HIST1H4H, HIST1H3H, HIST1H3B, HIST1H4C, HIST1H3C, HIST1H4D, HIST1H3D, HIST1H4E, HIST1H3E	6.41	1.13E-03	1.12E-03
GO:0032200~telomere organization	11	HIST1H4A, HIST1H3A, HIST1H4H, HIST1H3H, HIST1H3B, HIST1H4C, HIST1H3C, HIST1H4D, HIST1H3D, HIST1H4E, HIST1H3E	6.96	1.19E-03	1.19E-03
GO:0000183~chromatin silencing at rDNA	12	HIST1H4A, HIST1H3A, HIST1H4H, HIST1H3H, HIST1H3B, HIST1H4C, HIST2H3D, HIST1H3C, HIST1H4D, HIST1H3D, HIST1H4E, HIST1H3E	5.54	3.17E-03	3.16E-03
GO:0031047~gene silencing by RNA	21	PIWIL2, POM121, NUP205, NUP153, HIST1H4A, HIST1H3A, POM121C, XPO5, NUP62, HIST1H4H, HIST1H3H, HIST1H3B, HIST1H4C, HIST2H3D, NUPL2, HIST1H3C, HIST1H4D, POLR2J, HIST1H4E, HIST1H3D, HIST1H3E	3.23	3.17E-03	3.16E-03
GO:0051290~protein heterotetramerization	12	HIST1H4A, NLGN1, HIST1H3A, HIST1H4H, HIST1H3H, HIST1H3B, HIST1H4C, HIST1H3C, HIST1H4D, HIST1H3D, HIST1H4E, HIST1H3E	4.88	9.34E-03	9.31E-03
GO:0045815~positive regulation of gene expression, epigenetic	14	DEK, HIST1H4A, HIST1H3A, POLR1D, HIST1H4H, HIST1H3H, HIST1H3B, HIST1H4C, HIST2H3D, HIST1H3C, HIST1H4D, HIST1H4E, HIST1H3D, HIST1H3E	3.86	1.95E-02	1.94E-02

2083 genes exclusively downregulated in "S2":

Term	Count	Genes	Fold Enrichment	P value adjusted	FDR
GO:0030574~collagen catabolic process	27	COL18A1, COL15A1, COL12A1, FURIN, MRC2, ADAMTS2, ADAMTS3, CTSL, CTSK, COL10A1, PHYKPL, COL25A1, MMP2, COL1A1, MMP11, COL3A1, MMP14, COL1A2, COL4A2, COL5A1, COL4A1, COL4A4, COL6A2, COL5A2, COL6A1, COL6A3, COL8A1	3.82	6.53E-07	6.41E-07
**GO:0050919~negative chemotaxis**	17	SEMA6B, SEMA5A, SEMA6A, SEMA4D, SEMA3C, SEMA3D, ITGB3, SEMA6D, SEMA4C, SEMA3G, PDGFA, APOA1, SEMA3E, EFNA5, FLRT2, SLIT3, SLIT2	4.52	5.31E-05	5.21E-05
GO:0030206~chondroitin sulfate biosynthetic process	13	CSGALNACT1, CHST9, CHST7, CHPF, CSGALNACT2, BGN, XYLT1, DCN, VCAN, CHSY1, DSE, CSPG4, CHST3	4.70	1.07E-03	1.05E-03
GO:0007169~transmembrane receptor protein tyrosine kinase signaling pathway	27	BLK, LTK, FLT1, FLT4, IGF1R, ERBB2, NTF3, NPTN, FYN, PAG1, NTRK2, NTRK3, INSR, MATK, PILRB, PTK6, NGF, BMX, GFRA2, FGR, CD4, KIT, RAPGEF1, CSPG4, ROR2, FRK, ANGPTL1	2.54	2.19E-03	2.15E-03

GO:0035023~regulation of Rho protein signal transduction	24	PLEKHG4, FARP1, PLEKHG1, PLEKHG2, ARHGEF26, ARHGEF15, ARHGEF25, ARHGEF17, RASGRF2, PLEKHG5, PLEKHG6, ARHGEF16, KALRN, BCR, PREX2, FGD4, AKAP13, ABR, OBSCN, ALS2CL, FGD6, ALS2, ARHGDIB, ARHGEF6	2.68	2.59E-03	2.54E-03
GO:0007219~Notch signaling pathway	29	NOTCH2, APP, NOTCH3, NOTCH1, MAML2, NOTCH4, NRARP, KCNA5, DTX4, DLL1, DLL4, CDH6, APH1B, PLN, RPS19, HEY1, MYC, HES1, RPS27A, ZNF423, TIMP4, JAG2, TGFB1, JAG1, GOT1, ANXA4, NR0B2, HEYL, BMP2	2.28	6.17E-03	6.05E-03
GO:0007229~integrin-mediated signaling pathway	26	ITGB1, ITGB5, ITGB3, ADAMTS10, PRAM1, CTGF, ADAMTS13, ADAMTS3, ADAMTS1, ADAM23, ITGAX, ITGA1, ADAM33, APOA1, FGR, COL3A1, CEACAM1, ITGAD, ITGA10, ITGA11, ADAM12, ITGA8, ZYX, MYH9, CDH17, ITGA9	2.38	7.17E-03	7.04E-03
GO:0010951~negative regulation of endopeptidase activity	29	SERPINA11, ITIH5, SERPINA3, APP, CSTA, LXN, ITIH2, SERPINA10, SERPINE1, WFDC1, FURIN, SERPINA6, SERPINA4, SERPINA5, VTN, C5, TIMP2, SERPINH1, PI3, TIMP1, A2M, TIMP4, AGT, SPINT1, SLPI, CD109, COL6A3, HRG, LPA	2.17	1.25E-02	1.22E-02
GO:0001558~regulation of cell growth	22	IGFBP5, IGFBP4, HTRA3, IGFBP3, FAM107A, LTBP4, HTRA1, WFDC1, TMEM97, RASGRP2, CYR61, FBLN5, AGT, CTGF, RAB33B, CEACAM1, NOV, EPB41L1, AGTR1, PLCE1, IGFBP7, SGK1	2.49	1.26E-02	1.24E-02
**GO:0070098~chemokine-mediated signaling pathway**	20	CXCL6, CCL11, CCL21, CCL20, CXCR4, CXCL1, PPBP, CXCL3, CXCL2, CX3CL1, CXCL12, CXCR1, CCRL2, CXCR2, ACKR3, CCR6, CCL19, CCL18, CCR4, PF4	2.55	1.74E-02	1.71E-02
GO:0046069~cGMP catabolic process	6	PDE10A, PDE1B, PDE1A, PDE2A, PDE5A, PDE9A	7.75	2.34E-02	2.30E-02
GO:0010862~positive regulation of pathway-restricted SMAD protein phosphorylation	15	ACVRL1, LEFTY1, TGFB1, RBPMS, TGFB3, INHBB, INHBA, GDF6, ACVR1B, TGFBR1, BMP6, BMP5, INHBE, BMP2, ENG	2.83	3.58E-02	3.51E-02
GO:0046777~protein autophosphorylation	35	CAMK2B, FLT1, FLT4, LRRK2, PRKX, THY1, ACVR1B, CAMKK2, IGF1R, GRK5, ERBB2, EPHB1, EPHB4, PDGFRB, PDGFRA, NTRK2, DAPK1, NTRK3, INSR, IRAK3, PTK6, BMX, FGR, ERN1, BCR, FER, FES, PEAK1, CAMK4, KIT, PRKD2, SIK1, EPHA1, FGFR2, MAP3K12	1.84	3.73E-02	3.66E-02
GO:0045747~positive regulation of Notch signaling pathway	12	JAG2, LFNG, DLL4, TSPAN14, NOTCH1, JAG1, NOV, MFNG, KIT, HES1, DLL1, TSPAN10	3.29	3.80E-02	3.73E-02
GO:0018108~peptidyl-tyrosine phosphorylation	32	LTK, PKDCC, FLT1, DYRK2, FLT4, PDGFB, FGF1, CAMKK2, EFEMP1, ERBB2, NPTN, FYN, EPHB1, EPHB4, PDGFRB, PDGFRA, NTRK2, RIPK2, TIE1, NTRK3, INSR, DYRK1B, FGR, BCR, FER, FES, PEAK1, KIT, ROR2, EPHA1, FGFR2, EPHA3	1.89	3.99E-02	3.91E-02
GO:0051056~regulation of small GTPase mediated signal transduction	29	ARHGAP8, RASGRF2, ARHGAP15, ARHGAP6, KALRN, SIPA1L3, AKAP13, ABR, FGD4, SYDE1, ARHGDIB, A2M, SRGAP1, PLEKHG2, STARD8, RALGAPA2, ARHGEF17, PLEKHG5, ARHGEF16, ARAP2, ARHGAP29, ARAP3, RHOF, RHOD, ARHGAP24, BCR, OBSCN, RHOJ, ARHGEF6	1.96	4.21E-02	4.13E-02

793 genes exclusively downregulated in "S3":

Term	Count	Genes	Fold Enrichment	P value adjusted	FDR
**GO:0002479~antigen processing and presentation of exogenous peptide antigen via MHC class I, TAP-dependent**	23	PSMD11, PSMD13, HLA-A, HLA-F, PSMB10, HLA-E, PSMA5, PSMB6, PSMD6, PSMB4, PSMD7, PSMC3, PSMA1, PSMB2, PSMB3, PSMC1, PSMD2, PSME3, PSMD3, PSMB1, CD36, FCGR1A, B2M	8.86	7.03E-12	6.95E-12
**GO:0050852~T cell receptor signaling pathway**	29	PSMD11, PSMD13, GATA3, PSMB10, PSMB6, PSMD6, STK11, PSMB4, PSMD7, PSMB2, PSMB3, PSMD2, PSMD3, PSMB1, HLA-DPA1, HLA-DRB5, BTN3A1, NFKB1, PSMA5, IFNG, PSMC3, PSMA1, PSMC1, PSME3, HLA-DPB1, HLA-DRA, LCP2, HLA-DRB1, HLA-DQB1	4.75	1.50E-08	1.48E-08
GO:0038061~NIK/NF-kappaB signaling	19	PSMD11, PSMD13, PSMB10, NFKB1, PSMA5, PSMB6, PSMD6, PSMB4, PSMD7, PSMC3, PSMA1, PSMB2, PSMB3, PSMC1, PSMD2, PSME3, PSMD3, PSMB1, MAP3K14	6.99	7.26E-08	7.18E-08
GO:0006521~regulation of cellular amino acid metabolic process	17	PSMD11, PSMD13, PSMB10, PSMA5, PSMB6, PSMD6, PSMB4, PSMD7, PSMC3, PSMA1, PSMB2, PSMB3, PSMC1, PSMD2, PSME3, PSMD3, PSMB1	8.09	7.26E-08	7.18E-08
**GO:0060333~interferon-gamma-mediated signaling pathway**	19	CIITA, HLA-DRB5, PTAFR, HLA-A, HLA-F, HLA-E, IFNG, TRIM5, IRF2, HLA-DPB1, TRIM68, HLA-DRA, FCGR1A, GBP1, B2M, IRF9, HLA-DRB1, HLA-DPA1, HLA-DQB1	6.49	2.02E-07	1.99E-07
GO:0043488~regulation of mRNA stability	22	YTHDF2, PSMD11, PSMD13, PSMB10, PSMA5, PSMB6, PSMD6, PSMB4, PSMD7, PSMC3, PSMA1, PSMB2, PSMB3, PSMC1, PSMD2, EXOSC9, PSME3, MAPKAPK2, PSMD3, SERBP1, PSMB1, EIF4G1	5.18	4.56E-07	4.52E-07
**GO:0033209~tumor necrosis factor-mediated signaling pathway**	23	CD40, PSMD11, PSMD13, TNFSF12, TNFRSF9, PSMB10, TNFSF13B, PSMA5, PSMB6, PSMD6, PSMB4, PSMD7, PSMC3, PSMA1, PSMB2, PSMB3, PSMC1, PSMD2, PSME3, PSMD3, PSMB1, CARD14, MAP3K14	4.73	9.33E-07	9.23E-07
GO:0031145~anaphase-promoting complex-dependent catabolic process	18	PSMD11, PSMD13, PSMB10, PSMA5, PSMB6, PSMD6, PSMB4, PSMD7, PSMC3, PSMA1, PSMB2, PSMB3, PSMC1, PSMD2, PSME3, CDC27, PSMD3, PSMB1	5.53	4.06E-06	4.02E-06
**GO:0090263~positive regulation of canonical Wnt signaling pathway**	22	PSMD11, PSMD13, PSMB10, NLE1, CCAR2, NFKB1, ARNTL, PSMA5, PSMB6, PSMD6, PSMB4, PSMD7, PSMC3, PSMA1, PSMB2, PSMB3, PSMC1, PSMD2, PSME3, PSMD3, PSMB1, DVL1	4.45	4.06E-06	4.02E-06
**GO:0060071~Wnt signaling pathway, planar cell polarity pathway**	19	PSMD11, PSMD13, AP2A2, PSMB10, PSMA5, PSMB6, PSMD6, PSMB4, PSMD7, PSMC3, PSMA1, PSMB2, PSMB3, PSMC1, PSMD2, PSME3, PSMD3, PSMB1, DVL1	5.01	6.52E-06	6.45E-06
**GO:0002504~antigen processing and presentation of peptide or polysaccharide antigen via MHC class II**	9	HLA-DMA, HLA-DRB5, HLA-DMB, HLA-DPB1, HLA-DRA, HLA-DOA, HLA-DRB1, HLA-DPA1, HLA-DQB1	12.85	2.69E-05	2.66E-05
GO:0002223~stimulatory C-type lectin receptor signaling pathway	19	PSMD11, PSMD13, PSMB10, NFKB1, PSMA5, PSMB6, PSMD6, PSMB4, PSMD7, PSMC3, PSMA1, PSMB2, PSMB3, PSMC1, PSMD2, PSME3, PSMD3, PSMB1, PRKACB	4.39	4.67E-05	4.62E-05
GO:0043161~proteasome-mediated ubiquitin-dependent protein catabolic process	25	PSMD11, PSMD13, PSMB10, ARNTL, PSMB6, PSMD6, PSMB4, PSMD7, PSMB2, HECTD3, PSMB3, PSMD2, PSMD3, CDC27, PSMB1, RFFL, FBXL19, WWP2, CUL4A, PSMA5, PSMC3, PSMA1, PSMC1, PSME3, PLAA	2.99	5.75E-04	5.69E-04
GO:0000209~protein polyubiquitination	23	PSMD11, CDKN2A, PSMD13, UBE2J2, PSMB10, PSMA5, HERC5, PSMB6, PSMD6, PSMB4, PSMD7, PSMC3, PSMA1, PSMB2, PSMB3, PSMC1, PSMD2, PSME3, PSMD3, PSMB1, TPP2, TRIM69, UNKL	3.03	1.14E-03	1.13E-03

**GO:0090090~negative regulation of canonical Wnt signaling pathway**	21	PSMD11, PSMD13, PTPRO, MLLT3, PSMB10, PSMA5, PSMB6, CYLD, PSMD6, PSMB4, PSMD7, PSMC3, PSMA1, PSMB2, PSMB3, PSMC1, PSMD2, PSME3, PSMD3, PSMB1, DVL1	3.13	1.90E-03	1.88E-03
GO:0030816~positive regulation of cAMP metabolic process	5	CHGA, CXCL10, CXCL11, CXCL9, CXCR3	20.22	5.44E-03	5.39E-03
**GO:0019882~antigen processing and presentation**	11	HLA-DRB5, HLA-DMB, IFNG, HLA-DPB1, HLA-DRA, HLA-A, ULBP2, HLA-DRB1, HLA-DPA1, HLA-E, HLA-DQB1	4.85	9.26E-03	9.16E-03
GO:0051281~positive regulation of release of sequestered calcium ion into cytosol	8	CXCL10, CXCL11, CXCL9, CXCR3, CD19, XCL1, BDKRB1, DRD1	7.19	1.04E-02	1.03E-02
**GO:0038095~Fc-epsilon receptor signaling pathway**	20	PSMD11, PSMD13, PSMB10, NFKB1, PSMA5, PSMB6, PSMD6, PSMB4, MAPK8, PSMD7, PSMC3, PSMA1, PSMB2, PSMB3, PSMC1, PSMD2, PSME3, PSMD3, PSMB1, LCP2	2.73	1.65E-02	1.64E-02
**GO:0010818~T cell chemotaxis**	5	CXCL10, CXCL11, CXCR3, CCL3, PIK3CG	15.17	1.98E-02	1.96E-02
GO:0000165~MAPK cascade	25	SPTBN4, PSMD11, PSMD13, CSF2RB, PSMB10, PSMB6, PSMD6, PSMB4, PSMD7, PSMB2, PSMB3, PSMD2, PSMD3, PSMB1, CCL3, DUSP6, PSMA5, PSMC3, PSMA1, DLG4, PSMC1, PSME3, IL2RB, MAPKAPK2, MAP3K14	2.32	2.39E-02	2.36E-02
**GO:0019886~antigen processing and presentation of exogenous peptide antigen via MHC class II**	13	HLA-DRB5, AP2A2, HLA-DMA, HLA-DMB, AP1G1, HLA-DPB1, HLA-DRA, HLA-DOA, KIFAP3, CTSD, HLA-DRB1, HLA-DPA1, HLA-DQB1	3.43	4.00E-02	3.95E-02
**GO:0071346~cellular response to interferon-gamma**	10	GBP5, SYNCRIP, CIITA, CCL8, CCL4, CCL3, XCL2, XCL1, TLR3, HLA-DPA1	4.26	4.79E-02	4.74E-02

167 common downregulated genes in "S1" and "S2":

No significantly enriched pathways

578 common downregulated genes in "S1" and "S3":

Term	Count	Genes	Fold Enrichment	P value adjusted	FDR
GO:0042073~intraciliary transport	7	ICK, IFT74, TTC30B, TRAF3IP1, BBS12, TTC30A, HSPB11	14.81	7.42E-03	7.42E-03
GO:0005929~cilium	16	CEP104, DNAH12, NEK4, ARL6, CCP110, LRRC6, RPGRIP1L, WDR35, HSPB11, ICK, IFT74, TTC30B, BBS12, TTC30A, CEP250, CLUAP1	3.63	4.45E-03	4.41E-03

235 common downregulated genes in "S2" and "S3":

**Term**	**Count**	**Genes**	**Fold Enrichment**	**P value adjusted**	**FDR**
**GO:0006935~chemotaxis**	10	SPN, FOSL1, CYSLTR1, CCL23, XCR1, CCL22, C5AR2, ECSCR, CCL2, CCR7	6.37	7.65E-03	7.52E-03
GO:0070374~positive regulation of ERK1 and ERK2 cascade	11	CCL14, NRP1, CCL23, IL6, CCL22, C5AR2, KDR, CCL2, SPRY2, CCR7, TEK	4.89	1.76E-02	1.73E-02
GO:0001938~positive regulation of endothelial cell proliferation	7	PPP1R16B, NRP1, NRP2, EGR3, KDR, CCL2, TEK	7.89	3.52E-02	3.46E-02
GO:0010595~positive regulation of endothelial cell migration	6	NRP1, NRP2, KDR, TEK, ETS1, SASH1	10.14	3.74E-02	3.68E-02
**GO:0006955~immune response**	16	CCL14, CCL23, NRROS, CCL22, IFI6, TNFRSF10B, CFP, ETS1, SPN, IL6, OAS2, IRF8, CCL2, CCR7, HAMP, TRIM22	2.95	4.15E-02	4.09E-02

Supplementary table 8: Anti-mouse antibodies used for flow cytometry.

Antibodies	Clone	Vendor	Catalog number	Dilution
CD3e PerCP-Cy5.5	145-2C11	eBioscience	#45-0031-82	1:50
CD4 Pacific Blue	GK1.5	Biolegend	#100428	1:50
CD8 V500	53-6.7	BD Biosciences	#560776	1:50
NK1.1 Alexa Fluor 700	PK136	Biolegend	#108730	1:50
CD11b eFluor605	M1/70	eBioscience	#93-0112-42	1:50
CD25 APC	3C7	Biolegend	#101910	1:50
PD-1 PE/Dazzle 594	29F.1A12	Biolegend	#135227	1:50
CD19 PE/Cy7	6D5	Biolegend	#115520	1:50
CD69 APC/Cy7	H1.2F3	Biolegend	#104526	1:50
Granzyme B PE	NGZB	eBioscience	#12-8898-82	1:50
FOXP3 Alexa Fluor 488	MF-14	Biolegend	#126406	1:50
Live/Dead Fixable Blue	-	ThermoFisher	#L23105	0.73611111
